# Exploring the Relationship between Salivary Levels of TNF-α, *Lactobacillus acidophilus,* *Lactobacillus gasseri,* Obesity, and Caries in Early Childhood

**DOI:** 10.3390/pathogens11050579

**Published:** 2022-05-14

**Authors:** Lúcia Aparecida Federighi Pereira Leme, Karina Ferreira Rizzardi, Isis Bolsonaro Santos, Thaís Manzano Parisotto

**Affiliations:** Laboratory of Clinical and Molecular Microbiology, University São Francisco, Bragança Paulista, São Paulo 12916-900, Brazil; lucia.leme@usf.edu.br (L.A.F.P.L.); karina_f_r@hotmail.com (K.F.R.); isisbolsonaro@gmail.com (I.B.S.)

**Keywords:** salivary cytokines, dental caries, child, obesity

## Abstract

This research aimed to explore the relationship between tumor necrosis factor-α (TNF-α), *Lactobacillus acidophilus* (*L. acidophilus*), *Lactobacillus gasseri* (*L. gasseri*), obesity, and early childhood caries. After caries and obesity diagnosis based on the WHO criteria, 94 preschoolers were assessed. Unstimulated saliva was collected for analysis of TNF-α by the Milliplex system and for *L. acidophilus* and *L. gasseri* using real-time polymerase chain reaction (RT-PCR). In obese children, each unit increase in the body mass index (BMI), and the TNF-α levels was associated with a one-time increase in the number of decayed surfaces (*p <* 0.05). Meanwhile, in eutrophic preschoolers, the presence of *L. gasseri* and *L. acidophilus* was linked, respectively, to an increase of 3.04 and 1.59 times in the number of decayed surfaces (*p <* 0.05); in obese children, the presence of *L. acidophilus* was not significant (*p >* 0.05) and *L. gasseri* was shown as a possible protective indicator (RR:0.49–*p* < 0.05). In conclusion, TNF-α and BMI were connected to carious lesions only in obese preschoolers, suggesting that inflammation could be underscored when both pathologies are concomitant. The presence of both species of lactobacilli investigated was connected with early childhood caries in eutrophic children, whereas in obese children only *L. gasseri* was significant, and in an opposite way, reinforcing that obesity can modulate oral bacteria.

## 1. Introduction

Obesity is a serious non-communicable and complex disease, basically characterized by an imbalance between energy intake and energy expenditure, which can result in an accumulation of body fat that presents a health risk [1,2]. Among nutritional disorders, childhood obesity is identified as one of the most frequent problems and is considered a major public health issue [1,3], killing even more people than underweight in certain countries [4]. The number of obese people has nearly tripled since 1975, and in children under the age of five, the number reached 39 million in 2020 [4].

The increase in the prevalence of obesity can be explained in part by excessive consumption of high energy/ultra-processed food, and sedentarism. Furthermore, it has been shown that the composition of gut microbiota influences obesity onset in childhood [5]. Of interest, microbial dysbiosis and eating habits are related to another serious problem in the population, which affects more than 530 million children from all around the world: early childhood caries (ECC) [6].

In the carious process, the mineral loss from the tooth structure comes due to bacterial acidic action, a consequence of an imbalance of the oral microbiota in the cariogenic biofilm accumulated on the dental tissues, with an increase in acidogenic and aciduric species, such as lactobacilli [7]. 

*Lactobacillus* spp. are Gram-positive, catalase-negative, acidogenic, and aciduric oxygen-tolerant anaerobes, capable of performing both oxidative and fermentative metabolism [8]. The association between lactobacilli and ECC is well known [7,8,9]. However, in the majority of the clinical investigations, the exact specie of *Lactobacillus* persists unidentified, and consequently their exact role in the pathogenesis of caries was not entirely explored. 

Interestingly, it is suggested that some *Lactobacillus* spp. could act as new therapeutic possibilities in the treatment of obesity, favoring weight loss and contributing to a decrease in abdominal fat accumulation. While *Lactobacillus acidophilus* (*L. acidophilus*) administration in humans might lead to significant weight gain, *Lactobacillus gasseri* (*L. gasseri*) was associated with weight loss in obese individuals [10]. In addition, a systematic review of probiotics showed that the effect on body weight is strain-dependent and while *L. gasseri* strain BNR17 was able to reduce weight gain, *L. gasseri* L66-5 promoted weight gain [11].

The association between ECC and obesity has been discussed in the literature, and a recent metanalysis points to a positive relationship between these two pathologies [12]. Obesity, which is a chronic inflammatory disease, could increase the probability of oral disturbs [13] and might accelerate dental development [14,15] because body fat accumulation has been connected to hormonal and inflammatory changes [16]. A new concept of the biological function of adipose tissue has been reported, strengthening the idea that this tissue is not only associated with energy storage but is also a dynamic organ involved in numerous metabolic and physiological processes [17]. It is noteworthy that adipocytes can secrete cytokines (soluble proteins synthesized by cells that mediate intracellular communication), which, directly or indirectly, can increase the production of factors related to inflammation [18], contributing to the low-grade systemic inflammation in obesity.

In obese patients, the circulating levels of inflammatory cytokines are high, and some of them, i.e., tumor necrosis factor-α (TNF-α), can be identified in saliva [19]. TNF-α is secreted and expressed in the fatty tissue (by macrophages infiltration and activation due to expanded adipose cells) [20]. Its concentrations are linked to the adiposity stage and insulin resistance [21]. It is well known that TNF-α has a key role in the activation and recruitment of inflammatory cells and in the regulation of genes linked to inflammation [20,22]. Intriguingly, TNF-α mediates endothelial cell apoptosis, possibly influencing atherosclerosis [23], an obesity-associated comorbidity. Additionally, this cytokine may influence the genesis of obesity because it was suggested in animal experiments that the deletion of receptor 1 of TNF-α protects from diet-induced obesity through increased thermogenesis [24].

In ECC children, the salivary TNF-α levels before dental treatment were shown to be significantly increased in comparison to caries-free controls [25]. Additionally, in adolescents with carious lesions, levels of TNF-α in the whole saliva were found to be higher when compared with controls [26]. The exact function of this cytokine in the pathophysiology of ECC has not been studied and elucidated. However, it is well known that caries are intimately related to the accumulation of biofilm, commonly located close to the gingival margin or on occlusal surfaces, at the initial stages of the disease, and further inside cavities, being able to reach the pulp organ. Inside dental biofilms, bacteria might use numerous strategies to avoid the host systems of defense and can cause inflammation and damage. During the inflammatory process, the role played by TNF-α is of prime importance, as one of the first host responses to pathological threats [26]. This protein is mainly produced by monocytes/macrophages, and it is involved in models of cell signaling in general inflammation, participating in acute immune responses [21,27].

Even though the scientific literature presents papers attempting to reveal a possible association between obesity and TNF-α blood levels [28,29,30], few studies are investigating salivary levels [31,32,33] and none of them have focused on preschoolers, or jointly assessed the interplay between obesity, ECC, and TNF-α in the saliva. Scientific and technological advances have enabled analyzes of salivary components with increased specificity and sensitivity, characterizing a new era in terms of molecular diagnosis in the oral cavity. The use of saliva to monitor health status and illness is strongly desirable for well-being promotion, especially for young children, as it comprises an easy and non-invasive collection method.

Considering that the mouth seems to reflect the intestine condition regarding some bacteria [34,35] and that lactobacilli showed important fermentation activities in the physiological regulation of the intestinal microbiome, besides being important for caries development, studying *L. acidophilus* and *L. gasseri* in samples from the oral cavity was proposed in the present study. Moreover, no report has evaluated the presence of the species in biological material from the oral cavity, nor a possible influence of ECC on this connection.

Thus, this research aimed to explore the relationship between TNF-α, *L. acidophilus*, *L. gasseri*, and BMI and the presence of carious lesions in early childhood, considering eutrophic and obese children.

## 2. Results

This cross sectional-study examined individuals at preschool age from public schools in Bragança Paulista-SP, Brazil. This city has a population of about 170,000 people, with a human development index of 0.82 and fluoride levels in the tap water of 0.7ppm. Enrolled preschoolers were from similar socioeconomic backgrounds (low to mid) and spent the entire day at the kindergartens, where the meals were provided and the brushing performed with fluoridated toothpaste. The number of boys and girls included were, respectively, 42 and 52, and the mean age was 56.69 months (±10.27). Including early carious lesions, obese ECC children have about five primary surfaces of teeth affected by caries, whereas the eutrophic ones have seven, most of them active and untreated. Considering the body mass index, among eutrophic and obese preschoolers the mean and standard-deviations were 15.86 ± 0.86 and 21.45 ± 2.32, respectively.

The sample characteristics regarding TNF-α and lactobacilli in the population studied are displayed in Table 1. *L. gasseri* and *L. acidophilus* were more prevalent among children with ECC in the eutrophic condition, while in obesity these bacteria predominate in the caries-free preschoolers. With respect to TNF-α, the highest values were found when both disorders (obesity and caries) were concomitant in the same child, and a statistically significant difference was identified only between obese individuals with and without caries (*p <* 0.05).

The Poisson regression analysis, expressed by rate-ratio (RR), revealed the most significant risk indicators for ECC in obese and eutrophic children with respect to BMI, presence of lactobacilli, and levels of TNF-α assessed in the saliva of these individuals.

In Table 2, involving eutrophic children, the presence of *L. gasseri* and *L. acidophilus* were associated with an increase of 3.04 and 1.59 times, respectively, in the number of decayed, missing, or filled surfaces of teeth (*p <* 0.05). The body mass index and TNF-α levels were not significant in this model (*p >* 0.05).

Regarding obese children (Table 3), each unit increase in BMI and TNF-α levels was associated with an approximately one-fold increase in the number of decayed surfaces (*p <* 0.05). Additionally, in model 2, while *L. acidophilus* was not significant (*p >* 0.05), the presence of *L. gasseri* was an indicator of protection (RR:0.49–*p* < 0.05).

## 3. Discussion

Even though the etiology of obesity and ECC is complex and multifactorial, the microbiota is particularly connected with both diseases. Since the last century, *Lactobacillus* spp. (a beneficial part of several fermented foods) are recognized to be an important cariogenic bacterial group after mutans streptococci in the oral cavity, being able to be detected in plaque, dentine, and saliva samples [8,9,37,38]. Many studies involving lactobacilli species, in general, have shown their association with ECC [8,9,39], as the progressive destruction of mineralized tooth, tissues is a result of the action of organic acids produced by acidogenic bacteria. Moreover, LB are considered secondary invaders of pre-existing caries lesions [38] and specialists in caries progression [9]. In the present study, in the group of eutrophic children, the presence of *L. gasseri* and *L. acidophilus* were associated with an increase of 3.04 and 1.59 times, respectively, in the number of decayed surfaces (*p <* 0.05—Table 2). In line with these findings, *L. gasseri* was already identified in oral samples from children with ECC [8,39,40], as well as in the biofilm of children with ECC in the severe stage, especially in those harboring *Candida albicans* [41]. Concerning *L. acidophilus*, they were found in samples collected from carious sites from Tunisian children aged 4–12 years old [42], and recently it was suggested that the occurrence of LB species varied with the age of the child, and in the age group of 3–12 years, *L. acidophilus* were plentifully found over the carious surfaces, being linked to caries in children of central India [43].

Regarding obese children, while the presence of *L. acidophilus* was not significantly associated with caries, the presence of *L. gasseri* was a possible indicator of protection (RR:0.49-*p <* 0.05), showing that nutritional status (obesity) can influence the modulation of the behavior of these bacteria. However, the exact mechanism by which this occurs is still unknown and needs further elucidation. Interestingly, *L. acidophilus* was found to be absent in almost all biofilm samples collected from Brazilian preschoolers, independent of having caries or being caries-free [44]. However, in a previous study of the same group, when carious dentinal samples were collected, identification of *L. acidophilus* was significantly associated with the activity of the lesion in children aged 2–5 years [45]. This reinforces the fact that LB is supposed to be secondary colonizers of existing lesions and not requisite agents for carious lesions inception [8,9,46]. Previous investigations involving children at older ages (10–15 years) also showed that probiotics containing *L. acidophilus* (together or not with other microbes) are capable of promoting a statistically significant reduction in mutans streptococci, which is intimately related to the carious process initiation or the main caries pathogen [47,48]. This may explain the fact that the *L. acidophilus* was not associated with ECC in the group of obese children.

Precise information of the LB species is crucial to investigate their role in the disease course or even in health status, and long-term investigations assessing mouth colonization by probiotic bacteria are still necessary for possible future therapy application/development. Currently, it has been suggested that the oral microbiota may reflect the intestinal condition [34,35] because it is in the oral cavity that the digestion process begins, and a significant amount of exogenous microbes can be swallowed together with the food bolus, being able to resist the reduced stomach pH and reach the intestine. It is of prime importance to emphasize that the mouth is part of the human body and, therefore, changes occurring in it might be connected to systemic alterations, reinforcing a multidisciplinary and holistic approach for the patient`s health [13].

The inflammatory process is closely connected with dental caries and obesity. Although in tooth decay the inflammation occurs due to biofilm accumulation, chiefly adjacent to the gingival margin, which harbors numerous pathogenic microorganisms capable of producing toxins that lead to bleeding and swelling; in obesity, it is associated with the fat increase. In this respect, proinflammatory cytokines such as TNF-α play an important role in this process. TNF-α is a chemical mediator related to numerous pro-inflammatory actions and strongly induces the activation of a transcription factor, nuclear factor- κB (NF-κB), accountable for the regulation of genes connected with inflammation [20,22]. It is also a crucial component in obesity-linked insulin resistance by inhibiting insulin receptor signaling and the transportation of glucose in the target cells [21]. In the adipose tissue, TNF-α usually achieves high levels, and even though the exact mechanism leading to this increase is still unclear, positive biosynthesis autoregulation has been suggested, favoring the maintenance of elevated amounts in obesity [21]. However, TNF-α plays a key role in adipokine dysregulation [21,49]. Consequently, the adipose cells of obese individuals enhance adipokines production in response to direct TNF-α exposure, mediated by increased activity of the NF-κB pathway. This increased activity is connected with adipokine expression, adipocyte size, and insulin resistance [21].

Of interest, in the present study, there was a statistical difference in the levels of TNF-α between obese preschoolers with caries and those without (Table 1). Additionally, it was found that in obese children, each unit increase in BMI and TNF-α levels were associated with approximately one-fold increases in the number of decayed surfaces (RR: 1.18/1.13 *p <* 0.05-Table 3). The same did not happen in the group of eutrophic children, which was similarly demonstrated in the study of Ribeiro et al. [50], reinforcing the role of obesity in the pro-inflammatory state when associated with caries. This way, an increase of salivary TNF-α, triggered by obesity, may contribute to the aggravation of oral inflammatory conditions. A recent systematic review with meta-analysis, including most of the studies in the adult population, supported the idea that TNF-α appears to be reliably increased in the saliva of obese individuals compared to nonobese, and that salivary TNF-α levels might be a useful obesity marker [22].

It is imperative to underscore that most studies involving obesity assess TNF-α in serum, in the adult population or adolescents, demonstrating that obesity was allied to increased levels of TNF-α in the bloodstream [28,29,30]. The present study disclosed that saliva seems to be a good alternative to blood, involving easy and non-invasive collection, a significant advantage when young children were considered. To the best of our knowledge, the present investigation is the pioneer in assessing TNF-α in the saliva of such a young child population, interplaying obesity, and dental caries.

The oral cavity represents a highly informative niche, displaying numerous biological elements that might act as indicators of risk or protection for a disease. Even though the identification of *L. acidophilus, L. gasseri*, and TNF-α levels in the salivary fluid of children could be valuable to the planning of preventive strategies, providing insights to better control oral and systemic pathologies, such as caries and obesity, the present study has some limitations. One of them is that the cross-sectional design did not provide a causal effect between the parameters and the investigated diseases, and another is that bacterial species detection in the saliva could be less sensitive compared with detection in the biofilm tooth samples. Nevertheless, more complex methodologies involving sequencing and mass spectrometry, for example, will be able to provide more information about other bacterial species and other inflammatory cytokines. These gaps should be addressed in future studies.

## 4. Materials and Methods

### 4.1. Ethical Considerations

This study was approved by the Ethics Committee of the University São Francisco, USF (protocols: nº 46107015.2.0000.5514/42997115.4.00005514). Parents or guardians who agreed with the inclusion of their child in this research signed an informed consent form, following the Guidelines and Regulatory Norms of the National Health Council.

### 4.2. Sample

The present cross-sectional investigation included children of both sexes, from 3–5 years old, attending the largest public kindergartens in the urban area of Bragança-SP.

As part of a larger study encompassing 968 children [51], 94 children were selected to take part in the present study, after obesity diagnosis, according to a convenience sampling strategy. Of the 968 children examined, 78 were obese, and 44 of them voluntarily accepted to participate in the saliva collection. Therefore, these 44 were selected to take part in the present investigation, composing the obese group of children, being 23 with ECC and 21 free of caries. As for the eutrophic children, 600 were identified among the 968 initially assessed, and 50 were selected to take part in the present research, regarding the acceptance to join the saliva collection and the similarity of age (in months) and sex, regarding the obese preschoolers. Among the 50 eutrophics, 21 were free of caries and 29 had ECC.

Children taking antibiotics at the time of the saliva collection or using these medications in the 30 days preceding the saliva collection were excluded. Additionally, the ones with enamel defects or those with special needs were dismissed.

### 4.3. Assessing the Nutritional Status of Children through the Body Mass Index

To measure weight and height, a calibrated electronic scale and a non-extensible measuring tape attached to a wooden board at 90° degrees to the ground, together with a headboard, were used, respectively [34,35]. To measure height, children were placed erect, with feet and heels parallel and together, as well as with the Camper Plane parallel to the ground. The preschoolers were weighed wearing only light uniforms and no shoes, standing upright, in the center of the scale, with their arms at their sides. Clothes’ weight was subtracted from the final measure.

For the nutritional status assessment, the body mass index (BMI = weight (kg)/height^2^ (m)) was used, considering gender and age, adopting the instruments proposed by the WHO [52] as references. The following cut-off points were used for categorization of results:

1. Eutrophic: ≥ Z scores -2 and ≤ Z scores + 1 (3–5 years old); > Z scores -2 and ≤ Z scores + 1 (5 years old);
2. Obese: > Z scores +3 (3–5 years old); > Z scores +2 and ≤ Z scores + 3 (5 years old).

### 4.4. Assessing Early Childhood Caries through Clinical Examination

The children had their teeth cleaned and dried with gauze. The diagnosis of ECC was carried out using visual inspection, under head-set light, with a mirror and a ball-ended dental probe. The criteria of the WHO modified by the inclusion of active white spot lesions [9,53] was used. Thus, both cavitated and non-cavitated lesions were diagnosed as caries in this study. The examinations were performed by two dentists, calibrated by a gold standard examiner. At the beginning of the study, after the examiner had received all the theoretical and practical instructions regarding the criterion to be used, the inter-examiner agreement was calculated (Kappa:0.86) by reexamining about 10 children with a time interval of at least one week between examinations.

### 4.5. Saliva Collection

Saliva, without stimulation, was collected in disposable plastic cups and was immediately transferred to microcentrifuge tubes (1.5 mL), which were kept on ice during the collection period, using an icebox. In the Laboratory of Microbiology of the University São Francisco, the saliva was centrifuged at 11,000× *g* for 10 min under refrigeration (4 °C); the supernatant and the precipitate were frozen at −80 °C and used for the analysis of inflammatory cytokine TNF-α and *Lactobacillus* spp., respectively.

### 4.6. TNF-α Analysis

The concentrations of TNF-α present in saliva samples were measured using commercial kits (Merck—USA-Human Metabolic Hormone Magnetic Bead Panel) following the manufacturer’s recommendations. In summary, in a 96-well microplate, 200 μL of the assay buffer was pipetted per well and shacked for 10 min. After decantation, 25 μL of the standard solution or control were added to appropriate wells, and 25 μL of the assay buffer was added to the sample wells. Then, 25 μL of appropriate matrix solution was added to the standards and control wells, whereas 25 μL of neat samples solution was added to the sample wells. Beads were added to each well and incubated overnight at 4 °C. After that, well contents were removed, and the microplate was washed three times with 200 μL of the wash buffer. Fifty microliters of the detection antibodies solution were added and incubated for one hour. In addition, 50 μL of Streptavidin-Phycoerythrin reagent was added per well and incubated for 30 min. Well contents were removed, and microplates were washed 3 times with 200 μL of the wash buffer. Finally, 100 μL of the drive fluid was added, and the readings were performed on MagPix^®^ equipment (Luminex^®^ System-Merck, Jersey, NJ, USA).

### 4.7. Lactobacillus spp. Analysis through Real-Time Polymerase Chain Reaction (RT-PCR)

DNA extraction from saliva samples was performed using the Lucigen/Epicentre kit (MasterPure™ Complete DNA and RNA Purification Kit, Cat. #MC85200, Holliston, MA, USA), and the DNA concentration was measured using Biodrop equipment (Biodrop µLite Spectrophotometer, Biochrom US Inc., Holliston, MA, USA). Briefly, the salivary precipitate was resuspended in 500 μL of Tris/EDTA buffer for molecular biology, pH 8.0, (Sigma-Aldrich^®,^ St. Louis, MO, USA), and 150 μL were subjected to DNA extraction. For this process, 150 μL of a *lysis solution* containing proteinase K was added, and incubation for 15 min at 65 °C was performed. Samples were placed on ice for 3–5 min, then 150 μL of a *protein precipitation reagent* was added and centrifugation for 10 min (10,000× *g*) at 4 °C was conducted. Five hundred microliters of isopropanol were added to the recovered supernatant and mixed by inversion. Total nucleic acids were pelleted by centrifugation at 4 °C for 10 min. After that, the isopropanol was carefully removed without dislodging the pellet, which was rinsed twice with 70% ethanol. All of the residual ethanol was removed gently with a pipette, and the nucleic acids were resuspended in 35 μL of TE Buffer and used for identification of *Lactobacillus* spp. through RT-PCR.

Real-time assays were executed on a 7300 Real-Time System (Applied Biosystems, Foster City, CA, USA), using the SYBR Green Power up (Thermo Fisher Scientific, Carlsbad, CA, USA). Primer sequences used for *L. gasseri* (16S/23S rRNA genes) and *L. acidophilus* (16S rRNA gene) have already been defined in the literature (forward primer *L. gasseri*, 5′-AAGGGCGCACGGTGAATGCCT-3′ and reverse primer, 5′-TGCTATCGCTTCAAGTGCTT-3′ [54]; forward primer *L. acidophilus,* 5′-GATCGCATGATCAGCTTATA-3′ and reverse primer 5′-AGTCTCTCAACTCGGCTATG-3′ [44]). Primers were used to generate a 329- [54] and 124-bp amplicon [44]). A total of 1.5µL of the DNA extracted from saliva samples was used for each assay, together with 5 µL of SYBR Green Power up, 2.9 µL of H_2_O, 0.3 µL of forwarding primer, and 0.3 µL of the reverse primer. The real-time PCR program for the detection of *L. gasseri* included 50 °C for 2 min; 95 °C for 2 min; and 40 cycles of 95 °C for 15 s, 62 °C for 15s, and 72 °C for 1 min, while the detection of *L. acidophilus* included 50 °C for 2 min; 95 °C for 2 min; and 40 cycles of 95 °C for 15 s, 58° for 15 s, and 72 °C for 1 min.

Standard curves were performed to determine the absolute target quantity in the samples, using the following species: *L. gasseri* (ATCC 33,323-AF182721.1 NCBI Reference Sequence Database) and *L. acidophilus* (ATCC 4356-MT 645504.1 NCBI Reference Sequence Database), which were also used as positive controls. Considering the standard curves, the software (Sequence Detection Software version 1.3.1, Applied Biosystems, Foster City, CA, USA) interpolates the absolute quantity of the target in the test samples.

The critical threshold cycle was the one in which the detectable fluorescence was above the background (PCR cycle number < 39). Duplicates were performed in all RT-PCR assays: standards and DNA samples.

### 4.8. Statistical Analysis

Data were assessed in the Statistical Package for Social Science version 16.0 (SPSS Inc., Chicago, IL, USA), considering a 5% significance level, using descriptive statistics, Mann–Whitney test (to compare caries-free versus ECC in the obese or eutrophic groups of children), and Poisson regression analysis. A model evaluating the significant risk indicators for early childhood caries (main outcome dmf-s) in obese and eutrophic preschoolers was built, with respect to the investigated parameters: TNF-α, BMI, and lactobacilli. In this regression analysis, the rate ratio reflected the effect-size (3.00, large; 1.86, medium; 1.22, small) [36]. *L. acidophilus and L. gasseri* were assessed based on the presence or absence of the bacteria due to their low counts (*L. acidophilus* PCR cycle number range: 24.06–37.83; *L. gasseri* PCR cycle number range: 28.68–37.92) [9,35].

## 5. Conclusions

In conclusion, TNF-α BMI was connected to carious lesions only in obese preschoolers, suggesting that inflammation could be underscored when both pathologies are concomitant. The presence of both species of the lactobacilli (*L. acidophilus* and *L. gasseri*) investigated was connected with caries in eutrophic children, whereas in obese children only *L. gasseri* was significant and in an opposite way, reinforcing that obesity can modulate oral bacteria.

## Figures and Tables

**Table 1:** Sample characteristics in terms of TNF-α and lactobacilli.

	Eutrophic		Obese	
	Caries	Caries-Free	Caries	Caries-Free
	(*n* = 29)	(*n* = 21)	(*n* = 23)	(*n* = 21)
***L. gasseri* ^(presence)^**	19 (68%)	9 (32%)	10 (43%)	13 (57%)
***L. acidophilus* ^(presence)^**	19 (66%)	10 (34%)	7 (41%)	10 (59%)
**TNF-α**	7.00 ± 23.00	5.00 ± 10.00	9.00 ± 13.50 *	2.00 ± 2.00 *

%: percentage; Data inherent to TNF-α refer to: medians ± interquartile deviations; Lactobacilli were considered for presence and absence due to the low values found in the samples (PCR cycle number range: 24.06–37.92); Asterisks refer to the statistical difference by the Mann–Whitney test (*p <* 0.05), concerning caries versus caries-free inside the group of obese and eutrophic children.

**Table 2:** Early childhood caries indicators in eutrophic children.

	Early Childhood Caries in Eutrophic Children	
Parameters	Rate Ratio (95% CI)	*p*-Value
*Lactobacillus gasseri* (presence^reference^/absence)	3.04 (2.15–4.29)	<0.001 *
*Lactobacillus acidophilus* (presence^reference^/absence)	1.59 (1.20–2.11)	0.001 *
BMI	1.11 (0.94–1.32)	0.21
TNF-α	1.00 (0.99–1.01)	0.20

Poisson Regression Model. Main outcome: early childhood caries (number of decayed, missing, or filled surfaces of the teeth); * Statistically significant at *p* < 0.05. CI: confidence interval; n = 50; Omnibus Test: likelihood Ratio Chi-Square = 50.36; freedom degree 4; significance 0.000. Rate ratio was considered as a measure of effect-size: 1.22 (small); 1.86 (medium); 3.00 (large) [36]. Lactobacilli were considered for presence and absence due to the low values found in the samples (PCR cycle number range: 24.06−37.92).

**Table 3:** Early childhood caries indicators in obese children.

	Early Childhood Caries in Obese Children	
Parameters	Rate Ratio (95% CI)	*p*-Value
*Lactobacillus gasseri* (presence^reference^/absence)	0.49 (0.82–0.87)	0.014 *
*Lactobacillus acidophilus* (presence^reference^/absence)	0.71 (0.41–1.23)	0.223
BMI	1.18 (1.09–1.29)	<0.001 *
TNF-α	1.13 (1.09–1.61)	<0.001 *

Poisson Regression Model. Main outcome: early childhood caries (number of decayed, missing, or filled surfaces of the teeth); * Statistically significant at *p* < 0.05; CI: confidence interval; n = 44; Omnibus Test: likelihood Ratio Chi-Square = 106.99; freedom degree 4; significance 0.000. Rate ratio was considered as a measure of effect-size: 1.22 (small); 1.86 (medium); 3.00 (large) [36]. Lactobacilli were considered for presence and absence due to the low values found in the samples (PCR cycle number range: 24.06−37.92).

## Data Availability

The data supporting the findings of the study are available from the corresponding author, upon reasonable request.
